# Supplementary material for: The interactome of intact mitochondria by cross-linking mass spectrometry provides evidence for coexisting respiratory supercomplexes
Source: Mol Cell Proteomics. 2017 Dec 8;17(2):216–32. doi: 10.1074/mcp.RA117.000470 (PMC5795388; doi:10.1074/mcp.RA117.000470)
Supplement: Supplemental Data [file supp_RA117.000470_133922_0_supp_23978_fzffwf.zip › spectra_annotation/mito_DR_spectra_annotation/89-1-6-1-4-1.pdf]

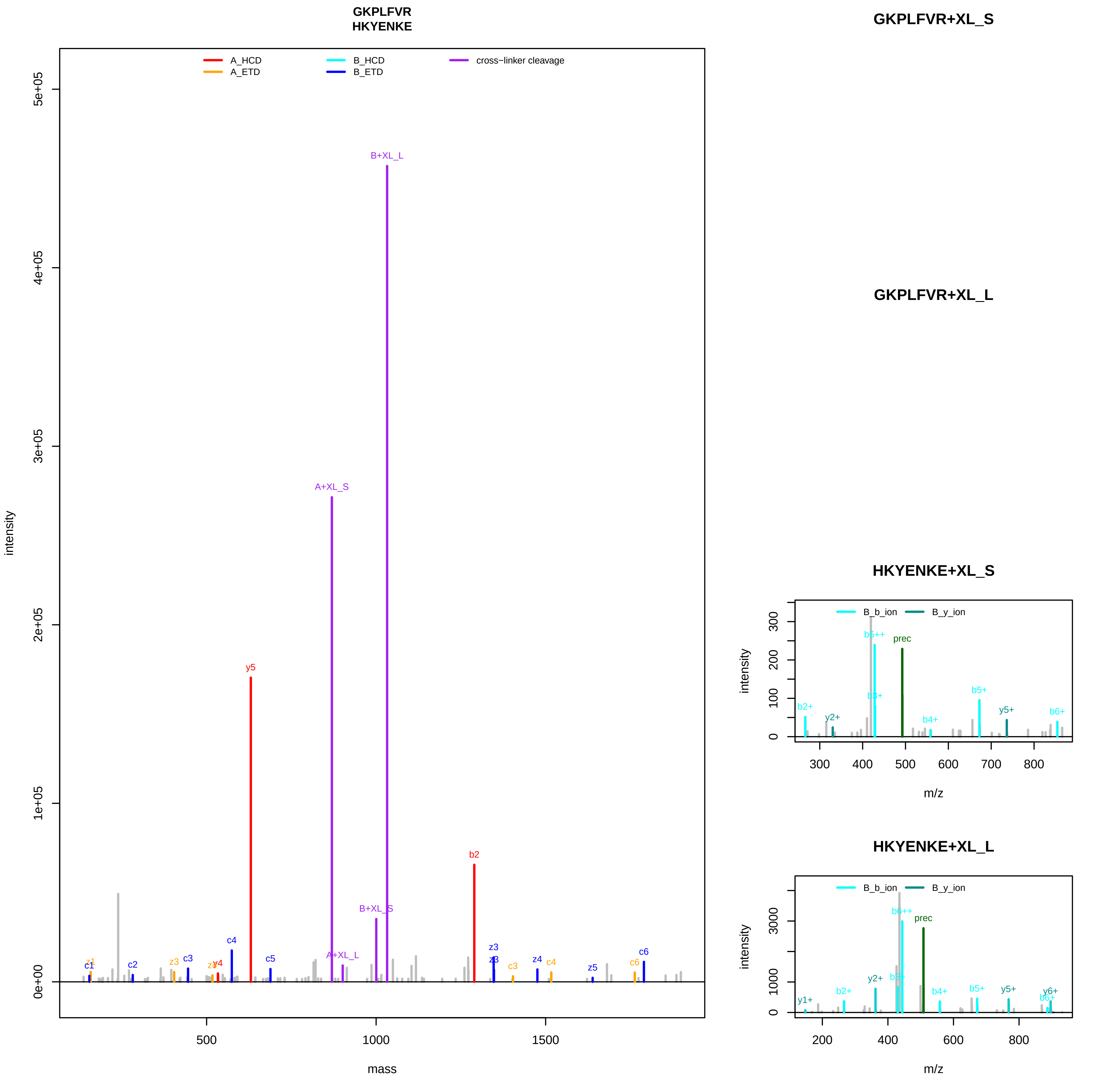
GKPLFVR
HKYENKE
A_HCD
A_ETD
B_HCD
B_ETD
cross−linker cleavage
intensity
mass
B+XL_L
A+XL_S
y5
b2
B+XL_S
A+XL_L
c1
z1
c2
z3
c3
z4
y4
c4
c5
z3
c3
z4
c4
z5
c6
c6
GKPLFVR+XL_S
GKPLFVR+XL_L
HKYENKE+XL_S
B_b_ion
B_y_ion
b6++
prec
b3+
b2+
y2+
b4+
b5+
y5+
b6+
m/z
HKYENKE+XL_L
b6++
prec
y2+
b3+
b2+
y1+
b4+
b5+
y5+
y6+
b6+
